# Bupivacaine injection for comitant horizontal strabismus: clinical and radiological results

**DOI:** 10.5935/0004-2749.20200032

**Published:** 2020

**Authors:** Umut Karaca, Halil İbrahim Altinsoy, Fatih Mehmet Mutlu, Osman Melih Ceylan

**Affiliations:** 1 Department of Ophthalmology, Faculty of Medicine, Isparta Suleyman Demirel Univercity, Isparta, Turkey; 2 Department of Ophthalmology, Gulhane Military Medical Academy, Ankara, Turkey; 3 Dunyagoz Eye Hospital, Istanbul, Turkey; 4 Department of Ophthalmology, Gulhane Education and Research Hospital, Ankara, Turkey; 5 Department of Ophthalmology, Yıldırım Beyazıt Education and Research Hospital, Ankara, Turkey

**Keywords:** Strabismus, Bupivacaine/administration & dosage, Oculomotor muscles, Magnetic resonance imaging, Estrabismo, Bupivacaina/administração & dosagem, Músculos oculomotores, Imagem por ressonância magnética

## Abstract

**Purpose:**

To report the outcomes of bupivacaine injection for the treatment of comitant horizontal strabismus and evaluate clinical effectiveness and associated radiological changes.

**Methods:**

This prospective observational clinical study was conducted on 10 patients with comitant horizontal strabismus of up to 40 prism diopters. Ophthalmologic examinations and three-dimensional orbital magnetic resonance imaging were performed pre and post-injection (at first, third, and 12^th^ months). A 4.5 ml of 0.5% bupivacaine was injected into the extraocular muscle under topical anesthesia using an electromyography in all patients.

**Results:**

The mean follow-up time at post bupivacaine injection and mean deviation at primary position were was 17 ± 2 months and 21.3 prism diopters, respectively. The mean changes in ocular alignment, enlargement of the cross-sectional area in the injected muscle, and volumetric enlargement were 7.7 PD, 12%, and 17% at the first year post-injection, respectively. No severe or persistent complication was observed. Ptosis and mydriasis were noted post-injection due to the anesthetic effects of bupivacaine but disappeared within 2 h post-injection.

**Conclusions:**

Bupivacaine injection improved eye alignment in small-angle horizontal comitant strabismus, effectively diagnosed with orbital magnetic resonance imaging to evaluate volumetric changes of the extraocular muscles. Further clinical studies with larger numbers of patients should be performed to define optimal dosages, concentration, and application method and dose-response relationship.

## Abstract (pt)

**Objetivo:**

Relatar resultados da injeção de bupivacaína para o tratamento do estrabismo comitante horizontal e avaliar sua eficácia clínica e as alterações radiológicas associadas.

**Métodos:**

Este estudo clínico observacional prospectivo foi realizado em 10 pacientes com estrabismo comitante horizontal de até 40 dioptrias de prisma. Exames oftalmológicos e ressonância magnética orbital tridimensional foram realizados pré e pós-injeção (no primeiro, terceiro e 12º mês). A 4,5 mL de bupivacaína a 0,5% foi injetado no músculo extraocular sob anestesia tópica usando eletromiografia em todos os pacientes.

**Resultados:**

O tempo médio de acompanhamento pós-injeção de bupivacaína e o desvio médio na posição primária foram de 17 ± 2 meses e 21,3 dioptrias de prisma, respectivamente. As alterações médias no alinhamento ocular, aumento da área da secção transversal no músculo injetado e aumento volumátrico foram de 7,7 PD, 12% e 17% no primeiro ano pós-injeção, respectivamente. Nenhuma complicação grave ou persistente foi observada. Ptose e midríase foram observadas após a injeção devido ao efeito anestésico da bupivacaína, mas desapareceram dentro de duas horas após a injeção.

**Conclusões:**

A injeção de bupivacaína melhorou o alinhamento dos olhos no estrabismo comitante horizontal de pequeno ângulo, efetivamente diagnosticado com ressonância magnética orbital para avaliar as alterações volumétricas dos músculos extraoculares. Outros estudos clínicos, com maior número de pacientes devem ser realizados para definir dosagens, concentração, método de aplicação e a relação dose-resposta.

## INTRODUCTION

Strabismus occurring after injecting a bupivacaine (BUP) as a local retrobulbar anesthetic agent for cataract surgery was first reported in the 1990s^(1-4)^. The physiopathology of this unexpected side effect was presented by Kushner^(5)^. Studies have revealed the occurrence of myotoxicity and necrosis post BUP injection, except in the basal lamina, nerves, and satellite cells (stem cell of myofibrils)^(6,7)^. With the release of growth factors from the damaged muscle fibers, satellite cells are activated to proliferate and form new muscle fibers^(8)^. This regeneration process results in hypertrophy of the affected muscle.

Scott et al. used this accidentally found effect of BUP as a new promising technique for the treatment of strabismus^(9)^. In this study, BUP was injected into the medial rectus (MR) or lateral rectus (LR) muscle of patients with comitant strabismus to analyze its effects on comitant strabismus and evaluate radiological findings.

## METHODS

Patients with comitant horizontal strabismus, who provided consent to receive BUP injection, were included in this study. Age at the initial examination, refraction, previous treatments (such as surgery or botulinum toxin injection), and best-corrected visual acuity were recorded. The deviation angles were measured by performing an alternate prism cover test at a distance of 6 m (far) and 0.33 m (near). Worth 4 dot and synoptophore were used to assess the binocular vision and sensory status, respectively.

A 4.5 ml of 0.5% BUP was injected into the extraocular muscle under topical anesthesia (proparacaine hydrochloride 0.5%): four exotropic patients received BUP via the MR and six esotropic patients via the LR, guided by electromyography (EMG). Patients treated with extraocular muscle surgery or botulinum toxin injection at least 6 months before the BUP injection were excluded from the study to prevent deviation instability.

All patients were examined on the first day, first month, third month, and first year post-injection. Complications occurring due to BUP injections and the deviation angle were recorded.

Magnetic resonance imaging (MRI) scans were obtained pre and post BUP injection (sixth hour, first month, third month, and first year). The pre-injection muscular status and the drug position within the muscle were evaluated. Muscular changes were noted on MRI examinations at the first and third months and first year.

Images obtained from all patients were acquired as vo lumetric (slice thickness of 1 mm) including both orbits using a 3.0 Tesla (T) MRI scanner (Philips Achieva X-series MR Systems; Best, The Netherlands). The 3D T1-weighted sequence obtained from the axial, coronal, and sagittal planes was used. Images were taken from the orbital apex to the anterior region, including the corneal surface. Patients were instructed to look up constantly at the primary position and keep their eyes closed. Otherwise, the examination was repeated.

The acquired images were transferred to the workstation (Vitrea 2^®^ version 4.1.2.0, Vital Images Inc., Minnessota, USA) and used to calculate the muscular area and volume. Using each slice on the axial and coronal planes, measurements were manually performed by drawing a line over the muscle from the origo at the orbit apex to the insertion point in the globe. Area and volume measurements were recorded in square centimeter (cm^2^) and cubic millimeter (mm^3^), respectively. The muscular area, the borders that had been drawn earlier, were automatically measured via the same software on the slice where the muscle was the widest. All measurements were carried out by two independent radiologists blinded to the details of this study. The mean value of these two independent measurements was used for the statistical analysis.

A commercially available SPSS 15.0 program was used for statistical calculations, and a p-value of <0.05 was considered significant in all statistical analyses. Wilcoxon and Mann-Whitney U analyses were conducted to compare the preand post-injection values, and Spearman’s correlation coefficients were used for the correlational analysis.

## RESULTS

All patient data are represented in table 1. Ten male patients with a mean age of 21.7 (20-27) years participated in this study and were divided into two groups: 6 with esotropia (Group 1) and 4 with exotropia (Group 2) The treatment outcomes were compared between these groups. None of the patients had undergone chemodenervation or strabismus surgery within the last 6 months.

**Table 1:** The details of all patients.

Patient	Age	Sex	Diagnosis	Previous surgery	VA/OD	VA/OS	Initial dev (∆)	Final dev (∆)	Change (∆)
1	20	M	XT	N	20/50	20/20	16	10	6
2	20	M	ET	N	20/20	20/20	25	18	7
3	23	M	XT	N	20/20	20/20	35	27	8
4	21	M	ET	+	20/20	20/20	18	16	2
5	22	M	XT	N	20/25	20/20	20	12	8
6	20	M	ET	N	20/20	20/100	20	16	4
7	23	M	ET	N	20/20	20/20	30	18	12
8	20	M	ET	+	20/40	20/20	25	14	11
9	21	M	ET	N	20/100	20/20	8	2	6
10	27	M	XT	N	20/20	20/20	8	2	6

**Figure 1:**
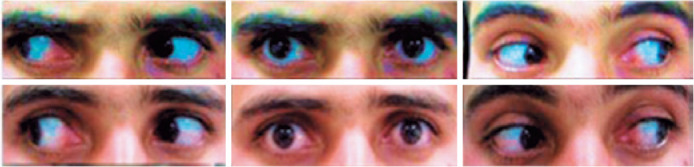
Pre-injection, 25 PD XT (up) and 16 PD residual XT at the 12^th^ month after injection (down).

The mean ocular alignment was 21.3 prism diopters (PD) at pre-injection and the mean alignment changes were 8.2 (p=0.005), 7.9 (p=0.004), and 7.8 (p=0.004) PD at the first, third, and 12^th^ months post-injection, respectively (Figure 1). The final mean ocular alignment changes were 8.6 and 6.5 PD in Group 1 and 2, respectively. The difference in ocular alignment changes was not significant between the two groups.

**Figure 2:**
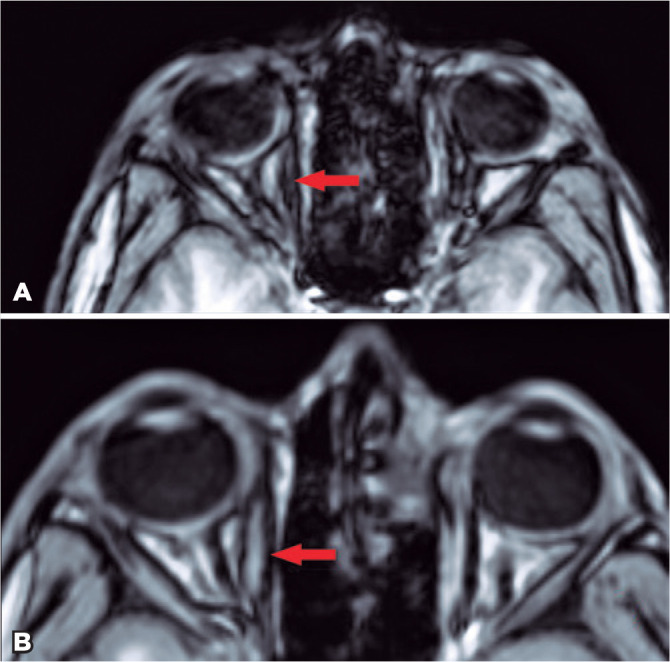
The MRI scans of the right medial rectus: before injection (A) and at the third month after injection (B).

The mean cross-sectional muscle area was 7.4 cm^2^ pre-injection and 8.6 cm^2^ within 6 hours post-injection. The mean muscle area was 8.5 cm^2^ (p=0.005) at the first and thirds months and 8.4 cm^2^ (p=0.005) at the 12^th^ month (Figure 2).

The mean muscle volume was 821 mm^3^ pre-injection and 976 mm^3^, 1020 mm^3^ (p=0.005), 995 mm^3^ (p=0.008), and 989 mm^3^ (p=0.008) at the sixth hour, first, third, and 12^th^ month, respectively.

Although the difference between ocular alignment and muscle volume changes were not statistically significant (p=0.067), these two parameters were found to be moderately negatively correlated (r=0.600). No persistent ocular complications, such as globe perforation, visual loss, persistent diplopia, proptosis, or retrobulbar hemorrhage, were noted at post-injection of BUP. Ptosis and mydriasis were also observed at post BUP injection due to its anesthetic effects but disappeared within 2 h post-injection. Common minor complications observed in these patients were temporary restriction in the field of action in the injected muscle and mild subconjunctival hemorrhage.

## DISCUSSION

Postoperative diplopia after administering retrobulbar anesthesia with BUP was reported in several patients in the late 1980s and 1990s^(10,11)^. This complication has been proposed to occur due to anesthetic myotoxicity. Animal studies showed that massive striated muscle fiber degeneration except the basal lamina, nerves, vas cular structures, and satellite cells is activated as myofibroblasts^(12,13)^.

BUP is the most potent myotoxic, aminoacyl anesthetic drug among all local anesthetic agents^(14)^. A single injection of BUP into the skeletal muscle has been shown to acutely cause muscle fiber cell lysis, membrane damage, and sarcomere dissolution. The remodeling process begins with supported myofibroblasts, and more contractile and larger myofibrils occur within 3-4 weeks^(15)^. Scott et al. reported a case of a 72-year-old woman with esotropia and diplopia initially treated with BUP injection^(9)^.

In another study, Scott et al. used different BUP doses and concentrations to determine the relationship between the dose and effect. In most patients, improved alignment was positively correlated with increased muscle volume; however, the small injection volume did not affect the posterior third of the muscle^(15)^. The largest case series of comitant horizontal strabismus treated with pharmacologic injection was reported by Debert et al. in 2016. They attempted to explain the physiological and biomechanical mechanisms of BUP injection and hypothesized that BUP induces hypertrophy causing an action on a shorter path^(16)^. In this study, we used the standard dose and concentration to minimize variability of results. Furthermore, EMG assistance facilitated the BUP injection process into the posterior third of the muscle.

Wutthiphan and Srisuwanporn reported that 75% of successful comitant and incomitant strabismic cases were administered with a standard dose and concentration^(17)^. Ocular alignment improved in 86.6% of comitant strabismus and 40% of incommitant cases. In our study, only comitant esotropic and exotropic cases were included to determine whether the BUP injection had any therapeutic effect. The BUP’s function depends on new, stronger contractile muscle fibers. Therefore, its effect on nerve paralysis, atrophic, or dystrophic muscle diseases is not observed in strabismus.

In this study, a statistical difference in the ocular alignment changes was not observed between the two groups (p>0.05). However, the BUP treatment seemed to provide better results for esotropic patients. Although the muscle volume in the LR does not differ from that in the MR, this situation may be due to the attainability of the MR muscle against the lateral rectus^(18)^.

MRI is the best noninvasive imaging technique for extraocular muscles^(19)^. In this study, Vitrea 2 workstation was used to measure the cross-sectional area and the volume of the drug-injected muscle. This allowed iden tifying changes in the area and volume at early and late phases of treatment. This is the first study in the literature to reveal the periodical radiological changes in the extraocular muscle after a BUP injection.

Previous studies have demonstrated that a good therapeutic response is related with the injection volume and amount of muscle fibers exposed to the drug^(15,20)^. The mean cross-sectional muscle area was 7.4 cm^2^ pre-injection and 8.6 cm^2^ within 6 h post-injection. The acute changes in the muscle area were evident in accurately placed injection and the importance of EMG assistance. In the first-year measurements, the mean increase was calculated as 12% for the cross-sectional area and 17% for the volume. The ocular alignment change was moderately negatively correlated with increased volume in the injected muscle (r=0.600). Similar results were reported in earlier clinical series. For example, Miller et al. identified the maximum mean increase in the cross-sectional area at the sixth month post-injection, which remained stable for 3 years^(20)^.

The results in this study, which are also supported by previous studies^(15-20)^, demonstrated that BUP injection into the weak muscle functionally improved in 6-8 PD based on satellite cell activation and regeneration. BUP injection into the extraocular muscle is a minimal invasive technique primarily performed in small-angle deviations, such as residual deviations. Further clinical studies are required to define the optimal dosage, concentration, and application method and elucidate the dose-response relationship.
